# Tranexamic Acid Reduces Total Blood Loss and the Amount of Stored Preoperative Autologous Blood Donation Needed for Adolescent Idiopathic Scoliosis Patients Undergoing Posterior Spinal Fusion

**DOI:** 10.7759/cureus.15488

**Published:** 2021-06-07

**Authors:** Takahiro Hideshima, Tsutomu Akazawa, Masahiro Iinuma, Yoshiaki Torii, Jun Ueno, Atsuhiro Yoshida, Hisateru Niki

**Affiliations:** 1 Department of Orthopaedic Surgery, St. Marianna University School of Medicine, Kawasaki, JPN

**Keywords:** tranexamic acid, total blood loss, autologous blood donation, adolescent idiopathic scoliosis, posterior spinal fusion

## Abstract

Introduction

There are few published studies on posterior spinal fusion (PSF) for adolescent idiopathic scoliosis (AIS) that have reported that the stored amounts of autologous blood donation (ABD) needed for the procedure were estimated by taking into account total blood loss (TBL). The aim of this study was to clarify the following clinical questions: (1) Does the use of tranexamic acid (TXA) reduce the TBL during PSF for AIS? (2) What volume of ABD should be stored to avoid allogeneic blood transfusions?

Methods

This study investigated 44 female patients who underwent PSF for AIS. A total of 33 patients underwent PSF without TXA (non-TXA group) and 11 patients underwent PSF with TXA (TXA group). TBL was determined by the hemoglobin (Hb) balance method calculated with circulating blood volume, Hb levels, hematocrit (Ht) levels before and three days after surgery, and the volumes of blood transfusions, including stored ABD. For the TXA patients, TBL was used to determine the appropriate amount of stored ABD and the number of ABD collections.

Results

The amount of TBL was lower in the TXA group compared to the non-TXA group. The mean required volume of stored ABD in the TXA group was 218.2 ± 577.3 mL, with a required maximum volume of 699.0 mL. The proportions of patients requiring allogeneic blood transfusion were as follows: 72.7% for those with no ABD collection, 45.5% for one ABD collection, and 0% for two ABD collections when TXA was used during surgery.

Conclusions

TXA reduced the TBL of patients undergoing PSF for AIS. The maximum amount of stored ABD needed was 699.0 mL. Allogeneic blood transfusion can be avoided by storing two ABD collections when TXA is used during the surgery.

## Introduction

Since tranexamic acid (TXA) has been reported to reduce perioperative bleeding, several papers reported its usefulness for total hip arthroplasty (THA), total knee arthroplasty (TKA), and spinal surgery [1,2]. TXA was also reported to reduce intraoperative blood loss during posterior spinal fusion for adolescent idiopathic scoliosis (AIS) [3,4]. In addition to measurable blood loss such as intraoperative and postoperative blood loss (bleeding from a drain), Foss et al. [5]. reported that there was unmeasurable blood loss during the perioperative period. They called the unmeasurable blood loss “hidden blood loss”. Intraoperative, postoperative, and hidden blood loss have been collectively defined as “total blood loss” (TBL). The methods for calculating TBL include the Gross equation, hemoglobin (Hb) balance method, Orthopedic Surgery Transfusion Hemoglobin European Overview (OSTHEO) formula, and the Hb-dilution method [6]. 

Preoperative autologous blood donation (ABD) is mainly used in posterior spinal fusion (PSF) for AIS to avoid allogeneic blood transfusion. However, there are few published studies on PSF for AIS that have reported that the stored amount of ABD needed for the procedure was estimated by taking into account the TBL. The aim of this study was to clarify the following clinical questions:

(1) Does the use of TXA reduce the TBL of PSF for AIS?

(2) What volume of ABD should be stored to avoid allogeneic blood transfusions?

We hypothesized that the use of TXA would reduce TBL and thus reduce the need for stored preoperative ABD.

## Materials and methods

Participants

Our institutional review board approved this retrospective study. The subjects were 52 consecutive AIS patients who underwent PSFs between August 2009 and July 2019. Eight patients were excluded. The reasons why the patients were excluded were as follows: five male patients, one patient with incomplete data, one patient with continuous injection of TXA, and one patient with anticoagulant therapy due to valvular disease. Finally, 44 patients were included in this research. The surgical procedure was pedicle screw fixation in ordinary methods. If the pedicle was small, this procedure was skipped, or we placed hooks or sublaminar bands with high molecular weight polyethylene tape. We performed inferior facetectomy (not including Ponte osteotomy) for the correction of scoliosis. After the operation, a suction drain was placed in the wound and removed on the second day after the operation. At the time of surgery, the participants’ mean age was 14.5 ± 4.4 years, mean height was 155.1 ± 11.2 cm, and mean weight was 44.3 ± 10.7 kg. The mean Hb of preoperative blood samples was 13.1 ± 1.6 g/dL and the mean hematocrit (Ht) was 39.4% ± 4.6%. The mean number of fused vertebral bodies was 9.1 ± 5.0 and the mean operative time was 306.6 ± 129.7 minutes. We divided the patients into two groups as patients with and without TXA. We started using TXA in surgery for AIS patients after July 2017, which is why some patients received TXA and others did not. There were 33 patients who underwent fusion without TXA from August 2009 to June 2017 (non-TXA group), and 11 patients who underwent fusion who received TXA from July 2017 to July 2019 (TXA group). In the TXA group, 1000 mg TXA was intravenously administered just before surgery and every five hours thereafter until the end of surgery. TXA was not administered after surgery. The following patient factors of both groups were compared: age, height, weight, preoperative Hb value, preoperative Ht value, number of fused vertebral bodies, operative time, preoperative proximal thoracic curve (PT curve), main thoracic curve (MT curve), thoracolumbar/lumbar curve (TL/L curve), and Lenke classification [7]. The volumes of intraoperative blood loss, postoperative blood loss, and TBL (including hidden blood loss) were also compared between both groups. 

Calculation of TBL

**Figure 1:**
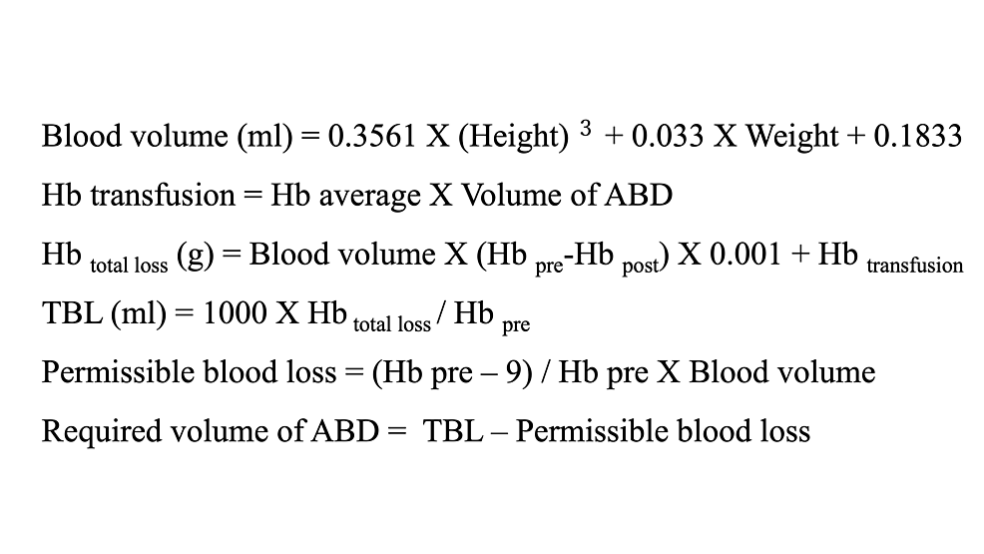
Calculation of total blood loss (TBL) with Hb balance method. Blood volume (ml): total blood volume before surgery Height (m): height before surgery Weight (㎏): weight before surgery Hb transfusion (g): volume of hemoglobin (Hb) in blood transfusion ABD (ml): autologous blood donation Hb average: average levels of Hb before ABD Hb pre (g/dl): level of Hb before surgery Hb post (g/dl): levels of Hb three days after surgery Permissible blood loss: blood loss keeping Hb levels more than 9 g/dl Required volume of ABD: appropriate volume of ABD

The Hb balance method [4] was used to calculate TBL. The method uses the circulating blood volume, Hb values before and three days after surgery, and the volume of transfused blood, which included ABD (Figure 1).

Preoperative autologous blood storage

**Figure 2:**
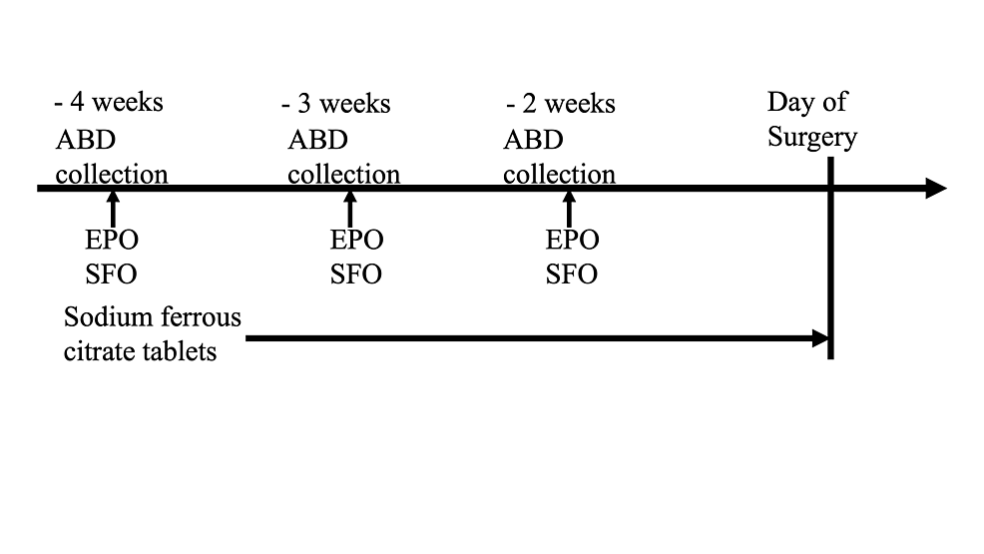
Schedule of preoperative autologous blood storage. ABD: autologous blood donation EPO: epoetin alfa injection (24,000 U) SFO: saccharated ferric oxide (Fesin®) (40 mg) Sodium ferrous citrate tablets: 100 mg/day from three weeks before surgery

The amount of preoperative ABD that was stored for each patient was based on guidelines for autologous blood storage [8]. For patients with a weight ≥ 50 kg, the amount of blood stored at one collection was 400 mL; and for patients with a weight < 50 kg, the amount of blood stored at one collection was 400 mL × (body weight/50 kg). For example, for a patient with a weight of 40 kg, 400 mL × (40/50) = 320 mL at one collection. ABD was collected and stored at 4, 3, and 2 weeks before surgery for a maximum of 3 collections. The patients were injected with 40 mg of colloidal saccharated iron oxide (Fesin®) and 24,000 IU of epoetin alpha at the time of collection and storage. The patients also took 100 mg of sodium ferrous citrate tablets daily PO (Figure 2). 

The amount of ABD and the number of ABD collections for the TXA group

**Figure 3:**
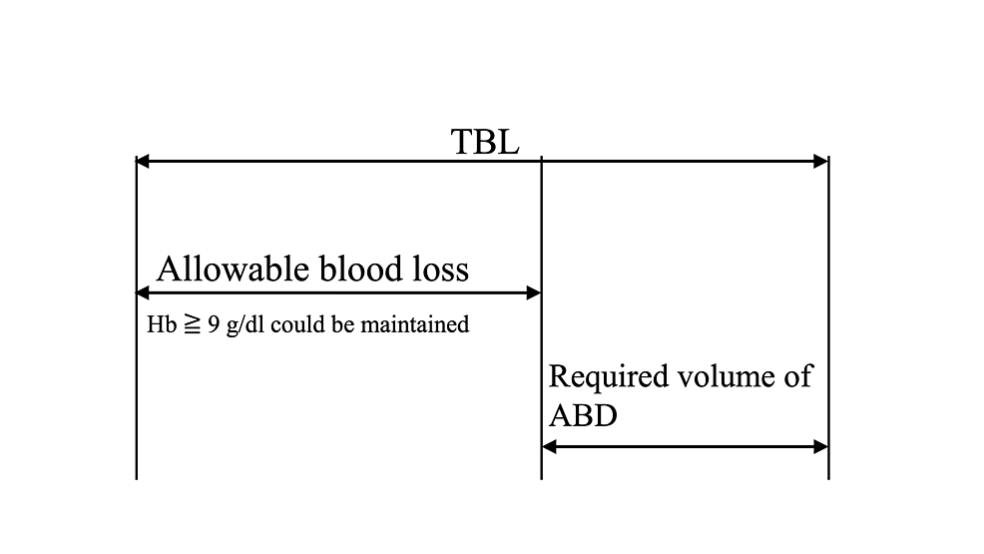
The difference between total blood loss (TBL) and allowable volume for blood loss was the volume of autologous blood donation (ABD).

The appropriate volume of ABD collected and stored and the number of ABD collections were calculated from the TBL for the TXA group. The "Guidelines for the Use of Blood Products" from the Ministry of Health, Labor and Welfare, Japan recommended Hb at 7-8 g/dL as the level indicating perioperative anemia and the need for transfusion [8]. To consider patient safety further, we used an Hb level of < 9 g/dL to indicate the need for transfusion during the perioperative period. The amount of blood loss that maintained a Hb level of ≥ 9 g/dL was defined as the allowable volume for blood loss (Figure 3). 

The proportion of patients requiring allogeneic blood transfusion was calculated for each ABD collection. In addition, the amount of ABD and the number of ABD collections needed to avoid allogeneic blood transfusion were calculated. 

Statistical methods

Values were expressed as means ± standard deviation. The Student T-test, Mann-Whitney U test, and Fisher exact test in EZR software (version 1.53, Saitama Medical Center, Jichi Medical University, Saitama, Japan) were used for analysis. The level of significance was set at < 5%. 

## Results

Patient demographic data 

The differences in age, height, weight, preoperative Hb level, preoperative Ht level, amount of ABD, or the number of fused vertebral bodies between both the groups were not significant. The operative time was significantly shorter for the TXA group than for the non-TXA group (Table 1). No patient sustained any major intra- or postoperative complications in either group. No adverse effects, such as venous thrombosis, headache, nausea, vomiting, or diarrhea, associated with the use of TXA were noted.

**Table 1:** Patient demographic data. Values were expressed as mean ± standard deviation. TXA: tranexamic acid, Hb: Hemoglobin, Ht: hematocrit, ABD: autologous blood donation.

	non-TXA group	TXA group	p-value
Age, years	14.5 ± 4.5	14.7 ± 3.9	0.73
Height, cm	154.5 ± 9.9	156.6 ± 13.8	0.30
Weight, kg	43.2 ± 10.5	46.3 ± 10.0	0.09
Preoperative Hb, g/dl	12.9 ± 1.6	13.5 ± 1.4	0.06
Preoperative Ht, %	39.1 ± 4.6	40.2 ± 4.3	0.16
Amount of ABD, ml	1042.6 ± 187.9	1104.5 ± 147.8	0.34
Number of fixed vertebral bodies	9.2 ± 5.1	8.5 ± 4.2	0.41
Operation time, min	319.6 ± 133.5	267.5 ± 75.6	0.02

X-ray images 

The differences in the preoperative PT, MT, or TL/L curves between both groups were not significant. Table 2 shows that there were no significant differences in Cobb angles on X-ray images. It highlights that there was no difference in the preoperative degree of deformity between the two groups. The Lenke classifications of the patients were as follows: non-TXA group included nice patients with type Ⅰ, eight with type Ⅱ, four with type Ⅲ, one with type Ⅳ, nine with type Ⅴ, and two with type Ⅵ scoliosis. The TXA group included six patients with type Ⅰ, two with type Ⅱ, one with type Ⅲ, and two with type Ⅴ scoliosis (Table 2). 

**Table 2:** X-ray images. Values were expressed as mean ± standard deviation. TXA: tranexamic acid, PT: proximal thoracic, MT: main thoracic, TL/L: thoracolumbar / lumbar.

	non-TXA group	TXA group	p-value
Preoperative PT curve, degree	27.6 ± 25.4	24.2 ± 14.8	0.42
Preoperative MT curve, degree	47.1 ± 33.4	45.5 ± 20.0	0.74
Preoperative TL/L curve, degree	37.1 ± 32.4	32.5 ± 22.3	0.40
Lenke classification (Ⅰ/Ⅱ/Ⅲ/Ⅳ/Ⅴ/Ⅵ)	(9/8/4/1/9/2)	(6/2/1/0/2/0)	

Blood loss 

**Figure 4:**
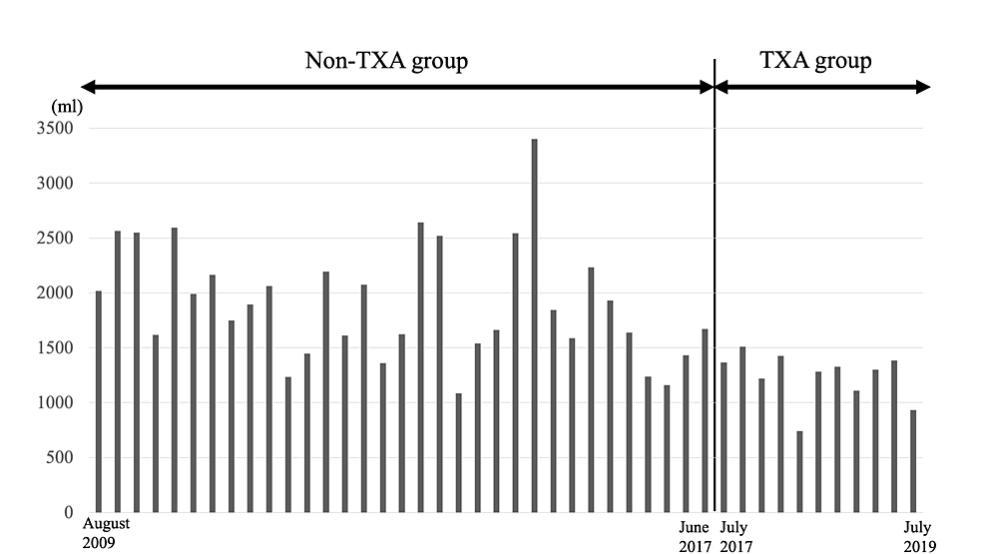
Total blood loss of all surgical cases.

The intraoperative volume of blood loss was significantly lower in the TXA group than in the non-TXA group. The difference in the volumes of postoperative blood loss between both groups was not significant. The volume of measurable blood loss (intraoperative plus postoperative blood loss) and volume of TBL were lower in the TXA group than in the non-TXA group (Table 3). A bar graph describing the total blood loss of all surgical cases is shown in Figure 4.

**Table 3:** Blood loss. Values were expressed as mean ± standard deviation. TXA: tranexamic acid, TBL: total blood loss.

	non-TXA group	TXA group	p-value
Intraoperative blood loss, ml	1267.7 ± 1402.3	331.6 ± 409.3	<0.01
Postoperative blood loss, ml	765.4 ± 577.2	555.5 ± 757.0	0.07
Measurable blood loss, ml	2033.1 ± 1481.9	887.1 ± 922.1	<0.01
TBL, ml	1905.3 ± 1031.7	1235.7 ± 434.6	<0.01

The amount of ABD and the number of ABD collections in the TXA group

The maximum volume of TBL in the TXA group was 1509.6 mL, and the volume of allowable blood loss was 1017.5 ± 259.5 mL. The mean required volume of stored ABD was 218.2 ± 577.3 mL, and the maximum value was calculated to be 699.0 mL. Based on these results, the proportions of patients requiring allogeneic blood transfusion were as follows: 72.7% for no ABD collection, 45.5% for one ABD collection, and 0% for two ABD collections. The required amount of ABD was 699.0 mL and the appropriate number of ABD collections was two. 

## Discussion

In our study, TXA seems to lessen ABD units that have to be stored preoperatively and thus decrease the allogenic blood transfusion rate. TXA is a drug that competitively inhibits the binding of plasmin and plasminogen to fibulin by binding to their lysine binding sites. TXA has been widely used for a long time in cardiovascular and gynecological surgery to reduce the amount of bleeding [9]. Ido et al. reported that the amounts of bleeding in total hip arthroplasty (THA) and total knee arthroplasty (TKA) procedures were reduced [1]. The amount of bleeding in spine surgery has been recently reported to be reduced when TXA is used [2], and several papers have reported that the use of TXA during posterior spinal fusion for AIS reduced intraoperative blood loss. Goobie et al. reported that the use of TXA reduced 27% of perioperative blood loss compared to a placebo group [10]. Bosch et al. reported that the transfusion rate dropped from 47% in the non-tranexamic acid cohort to 23% in the tranexamic acid cohort [11]. Eubanks reported that the use of antifibrinolytics in AIS surgery reduced blood loss and transfusion requirements [12]. Jones et al. reported that the use of TXA reduced the percentage of total volume lost versus no tranexamic acid in AIS patients who underwent PSF using a standardized blood loss measure [4]. Cheriyan et al. performed a meta-analysis of 11 papers reporting on 644 patients undergoing spine surgery who received TXA and found only one patient who developed an adverse effect myocardial infarction [2]. In our study, TXA reduced the amount of TBL in patients undergoing posterior spinal fusion for AIS, with no observed adverse effects. Although postoperative blood loss was not statistically different between the two groups, it might be due to the small sample size.

The reported methods of TXA administration include a single intravenous injection of 500 to 1000 mg or an intravenous drip infusion of 500 to 2500 mg [10,11]. In this study, intraoperative blood loss was significantly reduced in the patients receiving TXA, but the difference between the amounts of postoperative bleeding between the two groups was not significant. The effect of TXA might diminish after the procedure and did not affect postoperative blood loss. Since a comparative study based on the presence or absence of administered TXA after the surgery was not conducted, further investigation is needed. Furthermore, the question of which is more effective for AIS surgery, a single injection of TXA or continuous administration of TXA, remains unanswered [3,10]. 

There were several reports in terms of the relationship between the use of TXA and the operative time. Sui et al. reported that the use of TXA did not affect the operative time [3]. In contrast, Berney et al. reported that the use of TXA significantly shortened the operative time [13]. In our study, TXA significantly shortened the operative time. The reduction of intraoperative blood loss might affect operative time because less blood loss made surgeons see the surgical fields better.

Foss et al. reported on the existence of hidden blood loss in addition to the measurable blood loss (intraoperative plus postoperative blood loss) and stated that TBL was defined by the measurable and hidden blood loss [5]. The Gross equation, Hb balance method, OSTHEO formula, and Hb dilution method have been reported to be methods for estimating the TBL [6]. In this study, the measurable blood loss in the patients receiving TXA was 736.0 mL, whereas the estimated TBL by the Hb balance method was 1301.0 mL, a difference of 565.0 mL. In order to examine the exact amount of blood loss during the perioperative period, it would be accurate to use TBL. 

In a report on AIS surgery, Ikegami et al. reported that a mean volume of 700 mL of ABD was stored, and that allogeneic blood transfusion was avoided in 96% of patients [14]. Yumite et al. reported that a stored volume of 800 mL of ABD avoided allogeneic blood transfusion in all their patients [15]. Our study found that the maximum volume of required amount of stored ABD was 699.0 mL, and the appropriate number of ABD collection was two. 

This study has limitations. It was not a randomized clinical trial. With the exception of operative times, since the differences between the demographics of the two study groups were not significant, we think that the data from the two groups of this retrospective study were sufficient for making comparisons. Because this was a retrospective study, it was difficult to adjust the number of patients and preoperative Hb in each group. In our study, surgeries were performed in different periods. Because TXA had been reported to reduce perioperative blood loss in several studies, we could not perform a prospective study compared between the TXA group and the non-TXA group. TXA might reduce the operative time associated with intraoperative bleeding. Despite the number of fused vertebrae not being different, we thought that the reduction of intraoperative blood loss affected the operative time because less blood loss made surgeons see the surgical fields better. The mean main thoracic curve in our study patients was approximately 45°, indicating that this study did not include patients with severe scoliosis. The experience and learning curve of surgeons and minor improvements in surgical techniques and hemostatic devices were not considered in our study. These factors may affect perioperative blood loss. This study only included female patients because the number of male patients was too small for analysis. We plan to continue investigating a large number of additional patients.

## Conclusions

TXA reduced the TBL of patients undergoing posterior spinal fusion for AIS. The maximum amount of stored ABD needed was 699.0 mL. Allogeneic blood transfusion can be avoided by storing two ABD collections when TXA is used during the surgery.
